# Study on Fatigue Behavior and Life Prediction of Laser Powder Bed Fused Ti6Al4V Alloy at 400 °C

**DOI:** 10.3390/ma18245678

**Published:** 2025-12-18

**Authors:** Liangliang Wu, Ruida Xu, Jiaming Zhang, Huichen Yu, Zehui Jiao

**Affiliations:** Beijing Key Laboratory of Aeronautical Materials Testing and Evaluation, Aero Engine Corporation of China Key Laboratory of Materials Testing and Evaluation, AECC Beijing Institute of Aeronautical Materials, Beijing 100095, China

**Keywords:** additive manufacturing, selective laser melting, Ti6Al4V alloy, high cycle fatigue, fatigue life prediction

## Abstract

Additive manufacturing has huge development potential in the aerospace field. The hot-end components of aeroengines work in harsh environments, facing high temperatures and a demand for long service life. In this paper, high-cycle fatigue (HCF) tests of Ti6Al4V alloy at 400 °C by selective laser melting (SLM) under different stress ratios (−1, 0.1, 0.3, 0.5, and 0.8) were carried out, and the fracture surfaces were observed. The results show that the defects existing on the surface or subsurface are prone to become the origin of fatigue cracks. There is a large dispersion of the high-cycle fatigue life of the samples, especially at a low stress ratio. With the increase in the stress ratio, the fatigue failure area on the fracture surface gradually decreases, and the fracture surface gradually presents a mixed pattern of tensile endurance fracture and fatigue failure. Considering the influence of creep damage due to mean stress, models were established, respectively, for the fatigue behavior and time-related rupture behavior to predict fatigue life and conduct an assessment. Then, the two models were combined and the composite models were proposed using the linear damage law. Finally, the single fatigue model and rupture models, as well as the composite models, were evaluated, respectively, and compared with the actual fatigue life, and the best model was obtained for the high-cycle fatigue prediction of SLM Ti6Al4V at 400 °C.

## 1. Introduction

Additive manufacturing (AM) technology has the characteristics of simple processing, low cost, and high flexibility, which can realize the rapid forming of parts without molds, especially for new product development and small batch manufacturing of single pieces [1,2,3]. Selective laser melting (SLM) is a typical additive manufacturing technology, characterized by high forming precision and good surface quality, which can realize the direct precision forming of small and medium-sized complex components [2,4,5]. Ti6Al4V alloy is a common α+β titanium alloy, which has excellent comprehensive properties such as low density, high specific strength, good corrosion resistance, and high temperature resistance. It is one of the main materials for blades, disks, casing, and other parts of aeroengine fans and compressors, and its long-term service temperature can reach 400 °C [6,7].

Aeroengine components are subjected to a large number of alternating loads during the whole service life, and fatigue failure is the main failure mode. Consequently, the dynamic material properties must be sufficient to meet in-service loading and operational requirements. Many studies have shown that the fatigue properties of AM Ti6Al4V alloy are lower than those of traditional manufacturing alloys, and their fatigue properties are affected by many factors such as microstructure, sampling direction, and defects [8,9,10,11]. Leuders et al. [12] believed that the microstructure had an effect on the fatigue life of the SLM Ti6Al4V alloy, and after adjusting the microstructure by annealing treatment, the fatigue life of the material will increase. The research results of Chastand et al. [13] showed that defects were the main reason for the reduction in the fatigue properties of the SLM Ti6Al4V alloy, and the shape, size, and location of defects lead to the dispersion of fatigue life [14,15,16]. As for the anisotropy of fatigue properties, Nicoletto et al. [17] believed that due to the influence of the relative position of the loading direction and the defect long axis direction, the fatigue life of samples in the vertical direction was shorter. However, Chang et al. [18] found that horizontal samples were more likely to crack at *β* grain boundaries, resulting in shorter fatigue lives of materials. By optimizing process parameters [2,19,20] and with an appropriate heat treatment system [13,21,22,23], the number of defects can be reduced and the fatigue performance of the AM Ti6Al4V alloy can be improved. For aeroengine components, the high-cycle fatigue (HCF) stress-life (*S*-*N*) curve is the basis for “safety-life” design. Therefore, studying the fatigue behavior and understanding the fatigue properties of the AM Ti6Al4V alloy at service temperature are important bases for the life evaluation of the components.

Recent studies have shown that the fatigue life of additive manufacturing materials can be predicted by using fracture mechanics methods combined with the initial fracture size (or initial crack size) of the material [24,25]. Walker et al. [26] analyzed the defect size through the fracture analysis of the HCF test and equated it to the initial crack size. By using the linear elastic fracture mechanics method and combining the d*a*/d*N* − Δ*K* curve, the fatigue life of round bar samples was predicted. Shamir et al. [27] used the modified Hartman–Schijve formula to associate the small crack growth data with the long crack growth data based on the material constant obtained by long crack growth. The mean surface roughness of the original sample was equivalent to the initial damage size (EIDS), and the fatigue life of the three-point bending sample of WAAM Ti6Al4V alloy was predicted successfully by the method of fracture mechanics. However, in the existing reports, most of the fatigue life prediction methods for AM Ti6Al4V alloy are proposed based on fatigue test results at room temperature (RT) [14,28]. There is almost no research studying fatigue life prediction under a working temperature such as 400 °C. For the engineering application of the AM Ti6Al4V alloy, fatigue life prediction at working temperatures is indispensable.

In this paper, high-cycle fatigue tests were conducted to study the fatigue behavior of the SLM Ti6Al4V alloy at 400 °C, and fractography was performed by scanning electron microscopy (SEM) to obtain the fatigue fracture mechanism. Finally, a fatigue life prediction method was proposed based on the fracture morphology characteristics.

## 2. Materials and Methods

### 2.1. Materials and SLM Process

**Figure 1:**
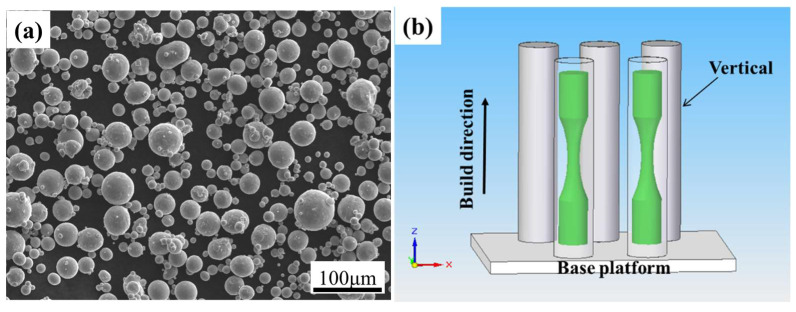
(**a**) Powder morphology of Ti6Al4V alloy; (**b**) schematic diagram of cylindrical blanks.

The experimental material was the Ti6Al4V alloy manufactured by the SLM process. The chemical composition of the alloy powder (Avimetal Powder Metallurgy Technology Co., Ltd., Beijing, China) is shown in Table 1. The powder morphology is spherical, as shown in Figure 1a, with the main particle sizes range from 20 to 60 μm. The model of SLM equipment used in AM is BLT-S300 (Xi’an Bright Laser Technologies Co., Ltd., Xi’an, China). The optimized parametersm which will further serve as potential process parameters for the AM of aeroengine components, were as follows: laser power *p* = 380 W, printing layer thickness *t* = 60 μm, scanning speed *v* = 1250 mm/s, and scanning spacing *h* = 120 μm. The scanning strategy in the AM process was bidirectional, and the scanning plane was the X–Y plane with the scanning direction angle 90° between two adjacent printing layers. The build direction was parallel to the *Z*-axis. The schematic diagram of the cylindrical blanks built by SLM are shown in Figure 1b. The overall AM process was completed under argon protection, with a chamber temperature of 35 °C, and then the blanks underwent annealing heat treatment. The process is as follows: 800 °C, vacuum holding for 2 h, and cooling in an argon environment.

### 2.2. Basic Information and Experimental Methods

**Figure 2:**
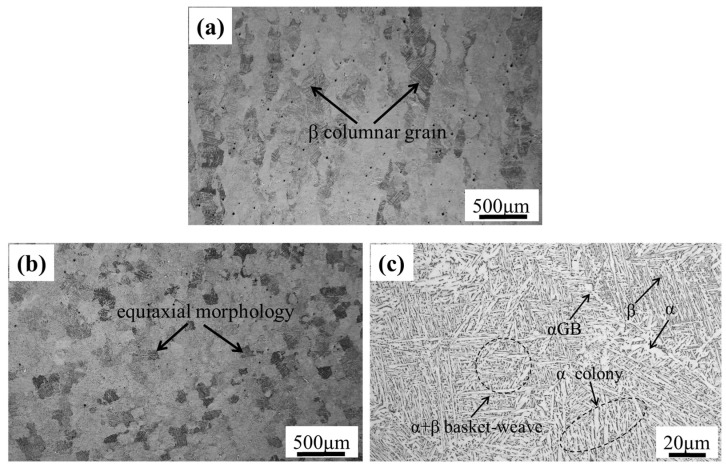
(**a**) Macroscopic morphology of the vertical plane; (**b**) macroscopic morphology of the horizontal plane; and (**c**) microscopic morphology.

The microstructure morphology of the SLM Ti6Al4V alloy is shown in Figure 2. The macroscopic morphology in the vertical plane, which is parallel to the *Z*-axis, shows a *β* columnar crystal structure elongated along the build direction, as shown in Figure 2a. In the horizontal direction (X–Y plane), which is perpendicular to the *Z*-axis, it exhibits an equiaxial morphology, as shown in Figure 2b. The structure of the columnar crystals and isometric morphology is composed of *α* lamellar structure and α+β basket-weave structure. The mechanical properties of SLM Ti6Al4V at 400 °C [29] were obtained from standard tensile tests [30], as shown in Table 2. It can be found that the tensile strength and yield strength of horizontal sample are higher than those of vertical sample. This may be caused by the anisotropic microstructure. In the process of tensile deformation, the hindering effect of grain boundaries on dislocation movement is the main reason for the improvement of material strength. The relative reduction in grain boundaries in the vertical plane due to the presence of β columnar grain undoubtedly weakens the resistance of dislocation movement, which is manifested as the reduction in strength. Conversely, equiaxial forms with smaller dimensions in the horizontal plane can effectively increase the number of grain boundaries, resulting in a higher strength [11].

**Figure 3:**
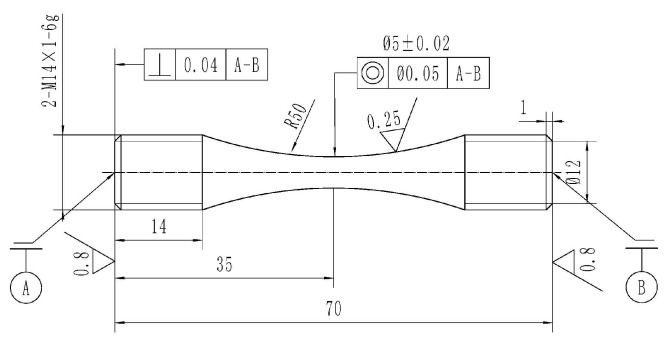
Shape and dimensions of the fatigue sample [32].

The high-cycle fatigue experiment was conducted according to test standard [31] on a high-frequency fatigue test machine. The shape and size of the sample is shown in Figure 3 [32]. After machining was completed, the arc section of the sample was polished until the surface roughness *R*_a_ was equal to 0.25. The experimental temperature was 400 °C, and the experiment was controlled by stress loading with a sine wave. The stress ratios were −1, 0.1, 0.3, 0.5 and 0.8, respectively, and the frequency was 100~120 Hz. For the samples that did not break after 10^7^ cycles, the experiment was stopped. After the fatigue test, the fracture surfaces of the samples were observed by Quanta FEG 450 scanning electron microscope (SEM) to analyze the fracture mechanism (Thermo Fisher Scientific, Waltham, MA, USA).

## 3. Results and Discussions

### 3.1. Experimental Results

**Figure 4:**
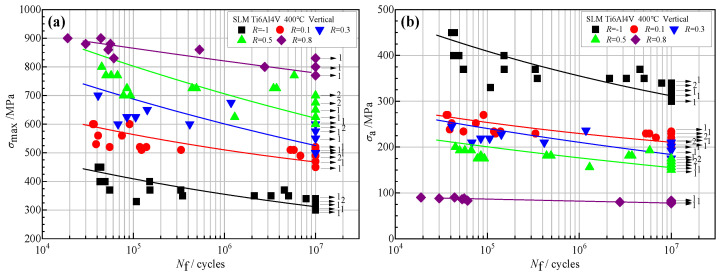
*S*-*N* data of the SLM Ti6Al4V alloy under different ratios. (**a**) *σ*_max_ vs. *N*_f_ and (**b**) *σ*_a_ vs. *N*_f_.

The results of the HCF test at 400 °C are shown in Figure 4 (the arrows in the figure represent the data points where no fracture occurred in 1 × 10^7^ cycles during the experiment, and the numbers behind represent the number of unbroken points at the corresponding stress levels). In the figure, the square symbol 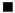 represents the fatigue data of the stress ratio −1, the round symbol 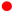 represents the fatigue data of the stress ratio 0.1, and the inverted triangle symbol 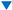; represents the fatigue data of the stress ratio 0.3. The triangular symbol 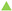 represents fatigue data with a stress ratio of 0.5, and the diamond symbol 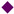 represents fatigue data with a stress ratio of 0.8.

To analyze the results under different stress ratios, this paper adopts the following three-parameter power function, Equation (1), for data fitting to obtain the fatigue *S*-*N* curve of the alloy.

(*σ*_max_ − *σ*_f_)*^m^*·*N*_f_ = *C* (1)

In the formula, *σ*_max_ represents the maximum stress, *σ*_f_ is the fatigue limit, *m* and *C* are material constants related to the stress ratio, and *N*_f_ stands for fatigue life.

The corresponding logarithmic expression is:

lg*N*_f_ = *B*_1_ + *B*_2_·lg(*σ*_max_ − *B*_3_) (2)

In the formula, the material parameters are *B*_1_ = lg*C*, *B*_2_ = −*m*, and *B*_3_ = *σ*_f_. The fitting parameters are shown in Table 3.

It was shown that in Figure 4a, at the same stress level *σ*_max_, the fatigue life of this alloy shows a gradually increasing trend with the increase in the stress ratio, which is mainly caused by the difference in stress amplitude *σ*_a_, as shown in Figure 4b. When *σ*_max_ is the same and with the increase of stress ratio, the stress amplitude *σ*_a_ (*σ*_a_ = *σ*_max_·(1 − *R*)/2) will decrease, and it will reduce the damage of the alloy during a single fatigue cycle. According to the principle of consistent total damage, the fatigue life of the alloy increases under higher stress ratio conditions. At the same time, it can also be seen from the figure that the fatigue life data of the alloy has the characteristic of high dispersion, and the degree of dispersion is related to the stress ratio to a certain extent. In high-stress ratios such as *R* = 0.8, the fatigue life dispersion is relatively small. Meanwhile, in low-stress ratios such as *R* = −1 and 0.1, the dispersion is relatively large, especially in the region near the fatigue limit stress level, and the difference in fatigue life between fractured and unfractured samples can reach several million cycles.

### 3.2. Fracture Surface Observation and Analysis

The fatigue performance of Ti6Al4V alloy by AM is jointly affected by multiple factors including defects, surface roughness, and residual stress. Studies have shown that the main defects in AM Ti6Al4V are porosity defects (including nearly spherical pores, irregular pores, and lack of fusion), with a small amount of spherical inclusions and microcracks caused by residual stress [13,17,18,20,21,22,26,33,34]. Due to the stress concentration at the defect site, the material is prone to develop into a crack source during the process of fatigue loading. Also, stress concentration can occur due to the high surface roughness with the “as-built” state, which indicates that there is no subsequent processing on the surface in AM Ti6Al4V. Yu et al. [22] compared the fatigue performance of SLM Ti6Al4V in its “as-built” state with that after different processing methods (turning, grinding, sandblasting after grinding, and polishing). The results showed that as the surface roughness decreased, the fatigue performance of the material increased successively. They believed that polishing was a more effective method to reduce surface roughness.

**Figure 5:**
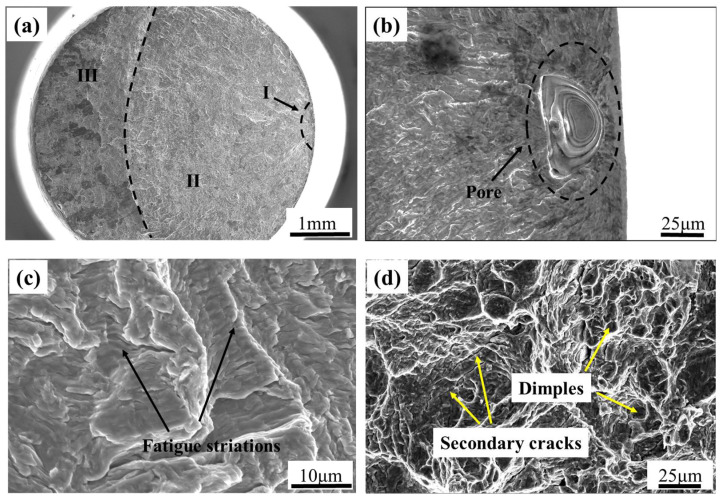
Fracture surface morphologies of a sample at *R* = 0.1 (*σ*_max_ = 490 MPa, *N*_f_ = 6.8 × 10^6^ cycles). (**a**) Macroscopic morphology; (**b**) crack nucleation region; (**c**) stable crack growth region; and (**d**) transient fracture region.

In this paper, the surface roughness of the specimens is uniformly polished to 0.25 to eliminate the influence of surface roughness on the fatigue performance of the specimens. And the fracture surfaces of the samples were observed and analyzed further to obtain the fracture mechanism of the material. The results show that there were three types of fatigue crack source characteristics: (1) surface and subsurface pore defects which come from the gas-entrapped during the AM process; (2) surface slips; and (3) internal pores, among which the first type of fatigue initiation feature is the most common, accounting for nearly 85% of the broken samples. Taking the fracture of a sample at *R* = 0.1 (*σ*_max_ = 490 MPa, *N*_f_ = 6.8 × 10^6^ cycles) as an example for illustration, as shown in Figure 5, the macroscopic morphology of the fracture surface shown in Figure 5a can be divided into three regions: the fatigue source region (I), crack propagation region (II), and transient fracture region (III). The crack source is a single crack source. The crack propagation region is relatively flat with multiple radial ridges visible, while the instantaneous fracture region is rough and uneven. Figure 5b shows the magnified morphology of the crack source area. The crack originates from the elliptical pores on the surface. Due to the easy occurrence of stress concentration at the defect site, the local stress increases, so the crack is prone to start from the defect site. Figure 5c shows the morphology of the crack growth zone, with distinct fatigue striations visible. At this point, the crack growth has entered a stable stage. Figure 5d shows the morphology of the final fracture zone, which has the characteristics of tensile fracture, with dimples of varying sizes and depths visible, showing a ductile fracture model.

**Figure 6:**
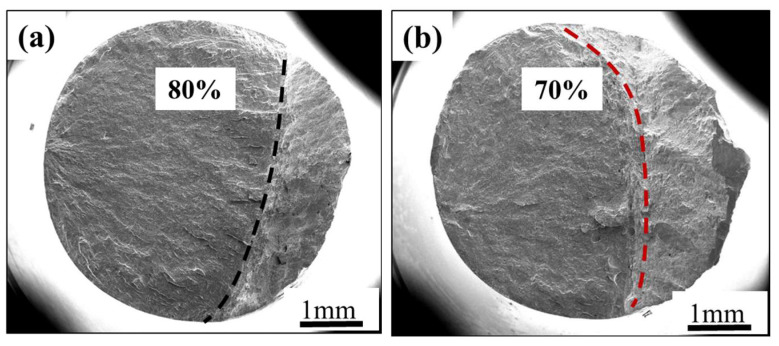

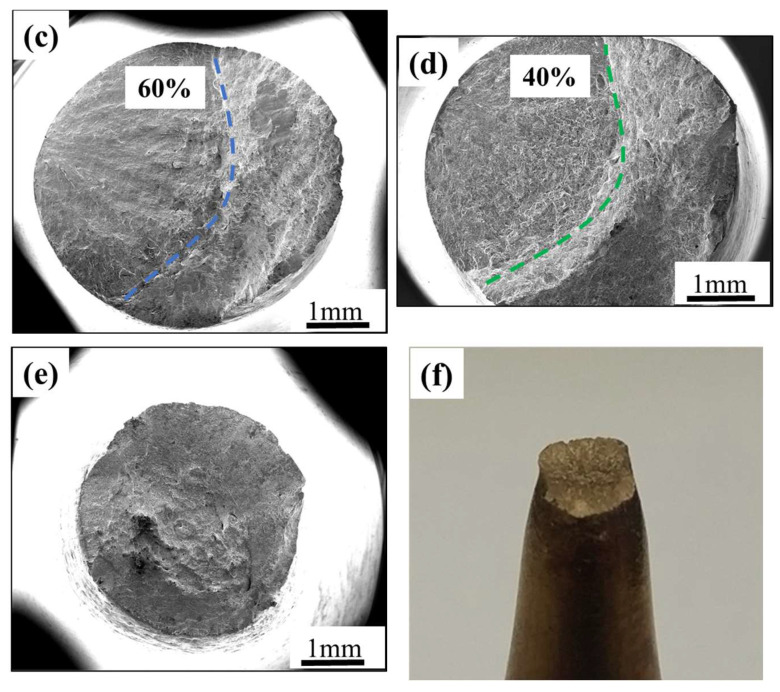
The macroscopic fracture morphology under different stress ratios. (**a**) *R* = −1, *σ*_max_ = 330 MPa, *N*_f_ = 1.08 × 10^5^ cycles; (**b**) *R* = 0.1, *σ*_max_ = 520 MPa, *N*_f_ = 1.39 × 10^5^ cycles; (**c**) *R* = 0.3, *σ*_max_ = 625 MPa, *N*_f_ = 8.5 × 10^4^ cycles; (**d**) *R* = 0.5, *σ*_max_ = 700 MPa, *N*_f_ = 9.3 × 10^4^ cycles; (**e**) *R* = 0.8, *σ*_max_ = 830 MPa, *N*_f_ = 6.1 × 10^4^ cycles; and (**f**) side profile of the fracture surface.

The macroscopic morphologies of the fracture surface when the fatigue life is approximately 1 × 10^5^ cycles under different stress ratios are shown in Figure 6. Figure 6a shows the macro fracture morphology of the sample *R* = −1. Through the observation of the fracture surface, and based on the discussion in Figure 5 above, it can be known that the fatigue failure area accounts for approximately 80% of the total fracture area. By analogy, the proportions of fatigue failure zones on the fracture surface when the stress ratios are 0.1, 0.3, and 0.5 can be obtained, which are 70%, 60%, and 40%, respectively, as shown in Figure 6b–d. As for *R* = 0.8, the macro fracture morphology of the sample is shown in Figure 6e, and the fatigue failure area of fracture morphology is not as obvious as that under other stress ratio conditions. The fracture has necking deformation, as shown in Figure 6f, and its shape is a cup-cone, with coarse fiber in the middle and a smooth shear lip around it. The fracture morphology is characterized by both fatigue failure and static tensile endurance fracture.

From the observation of the fracture morphology, it can be found that as the stress ratio increases, the fatigue failure area of the sample’s fracture surface gradually decreases. Especially at high stress ratio such as 0.8, the fatigue failure characteristics are no longer obvious, and instead, tensile endurance fracture characteristics take their place. This transformation is mainly influenced by the mean stress. When *R* = −1, 0.1, and 0.3, the mean stress is low; most of the fatigue cracks originate from the defects on the subsurface of the fatigue samples, and the fracture morphology shows obvious fatigue failure characteristics. However, under the condition of high stress ratio, such as *R* = 0.8, the mean stress is higher than the low stress ratio. On the one hand, during the fatigue loading process, the high mean stress will continuously load, causing the sample to be subjected to a fixed tensile load. On the other hand, at 400 °C, the continuous high mean stress may also lead to the occurrence of creep damage, so that at high stress ratios, the fracture surface of the sample shows a mixed mode of fatigue failure characteristics and tensile endurance fracture characteristics. Generally speaking, Ti6Al4V has sufficient creep resistance below 400 °C, and during use, the failure mode is generally fatigue failure. Above 400 °C, with the operating temperature increases, creep performance [35] increasingly becomes a key factor restricting the performance and service life. Therefore, for the life prediction of high-cycle fatigue of SLM Ti6Al4V, the influence of mean stress or creep damage generated under high mean stress should be considered.

## 4. The Fatigue Life Prediction

### 4.1. Fatigue and Rupture Model Analysis and Life Prediction

#### 4.1.1. Analysis of Fatigue Model and Fracture Model Related to Time

The discussions in Section 3.2 indicate that under the condition of lower stress ratio, the fracture mode of the sample is dominated by fatigue failure characteristics. Under the condition of high stress ratio, the fracture of the sample is dominated by the mixed mode with the characteristics of fatigue failure and tensile endurance fracture. Therefore, the prediction of high-cycle fatigue life under different stress ratios needs to consider the fatigue model and the time-dependent rupture model.

Fatigue Model:

As for the high-cycle fatigue model, if three or more *S*-*N* curves with different stress ratios have been obtained, the equivalent stress (*σ*_eq_) model can be used for description. The fatigue life model [36] is as follows:

lg*N*_f_ =*A*_1_ + *A*_2_·lg*σ*_eq_ (3)

For the calculation of equivalent stress *σ*_eq_, the method proposed by Walker [37] is generally adopted:

*σ*_eq_ = *σ*_max_ (1 − *R*)^(1−*w*)^ (4)

where *σ*_max_ is the maximum stress, *R* is the stress ratio, and *w* is the material parameter, which is called the Walker parameter. This method is referred to as the Walker equivalent stress model or Walker Model.

Rupture Models:

Wright et al. [36] introduced two time-affected fracture models to describe the fracture in which stress changes periodically with time. The first is a power function, as shown in Equation (5).

*t*_r_ = *K*_r_ (*σ*_eq_)*^n^* (5)

where *t*_r_ is the time taken to break, expressed in seconds (s), and *K*_r_ and *n* are the test fitting parameters.

When the sample is subjected to cyclic load, the alternating stress component and the mean stress component simultaneously affect the fracture of the sample, so they need to be treated to apply Equation (5). The simplest method is to assume that the alternating stress component has no effect on the fracture and only consider the effect of the mean stress (*σ*_mean_). Therefore, the value of *σ*_eq_ can be determined by the following formula:

*σ*_eq_ = *σ*_mean_ (6)

This method is referred to as Rupture Model *a* below.

The second fracture model integrates the fracture damage during the whole test process and considers the different effects of the alternating stress component and the mean stress component. The whole cycle is divided into small time increments, and it is considered that the alternating stress component and mean stress component are a certain value in the infinitesimal time increment, and the damage caused by it has a cumulative effect on the final fracture. Throughout the fracture life, the damage accumulation caused by countless infinitesimal time increments of internal stress leads to the final fracture.

For the sine wave load waveform, there is

*σ*(*t*) = *σ*_mean_ + *σ*_a_·sin(2π*t*/*τ*) (7)

When the break time is *t*_r_, the damage accumulation *D*_R_ of the final break at the expected lifetime *τ* should be 1:

(8) $$D_{R}=1=\int_{0}^{\tau }dt/t_{r}\left(\sigma \left(t\right)\right)$$

The time or number of cycles of the final break can be calculated by the above equation (*N*_f_ = *t*_r_ × *f*, where *f* is the frequency).

In this method, if the compressive stress state *σ*(*t*) < 0 occurs when *R* < 0 affects the final fracture, the cumulative damage caused by the stress load in the whole cycle is calculated, and the method is called Rupture Model *b*.

If it is believed that the compressive stress state *σ*(*t*) < 0 occurs when *R* < 0 has no effect on the final fracture; that is, the damage accumulation during the period of compressive stress state is ignored, and this method is called Rupture Model *c*.

For the test *R* > 0, the results of Rupture Models *b* and *c* are consistent because there is no compressive stress state. In addition, since there is no analytical solution to Equation (8), numerical integration is needed in the calculation of Rupture Models *b* and *c*. See Appendix A for the calculation process.

#### 4.1.2. Fatigue Life Prediction by Fatigue and Rupture Models

The fitting parameters of each prediction model are shown in Table 4 and Table 5.

**Figure 7:**
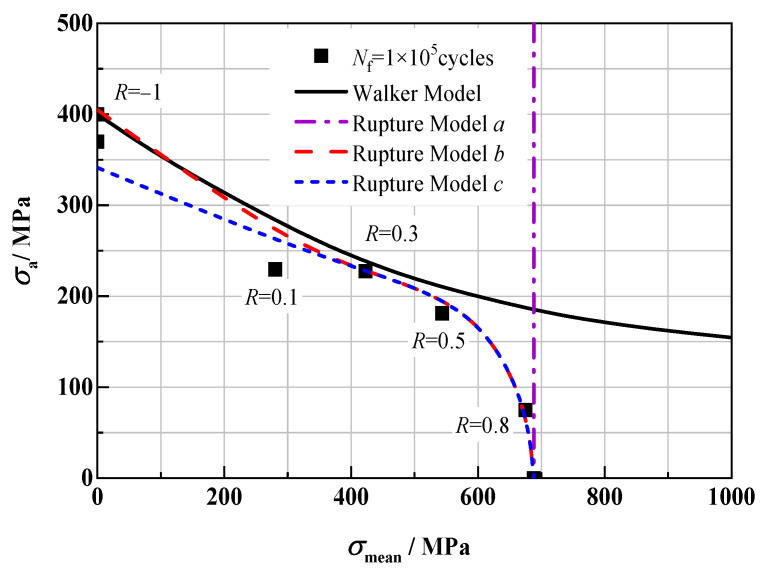
Equal life curve (*N*_f_ = 1 × 10^5^) of different life models.

Figure 7 shows the equal life curve of the above models at 1 × 10^5^ cycles. The Walker Model can fit the test data points well at a low stress ratio (*R* < 0.5), but at a high stress ratio (*R* > 0.5), the maximum stress of the fitted data points is far greater than the actual situation, and the fitting results are not ideal. Rupture Model *a* can fit the condition of high mean stress well, but with the decrease in *R*, the fitting result deviates greatly from the actual test data points, and it cannot be used to predict the fatigue life under the condition of *R* < 0.8 at all. Considering the effect of alternating load, the predicted results of Rupture Model *b* are in good agreement with the measured values. The fitting result of Rupture Model *c* is completely consistent with that of Rupture Model *b* under the condition of positive stress ratio, but under the condition of *R* < 0, the stress level of the fitting point is obviously low, and the fitting result is poor. Relatively speaking, among the above four models, Rupture Model *b* has the best predictive ability relatively.

**Figure 8:**
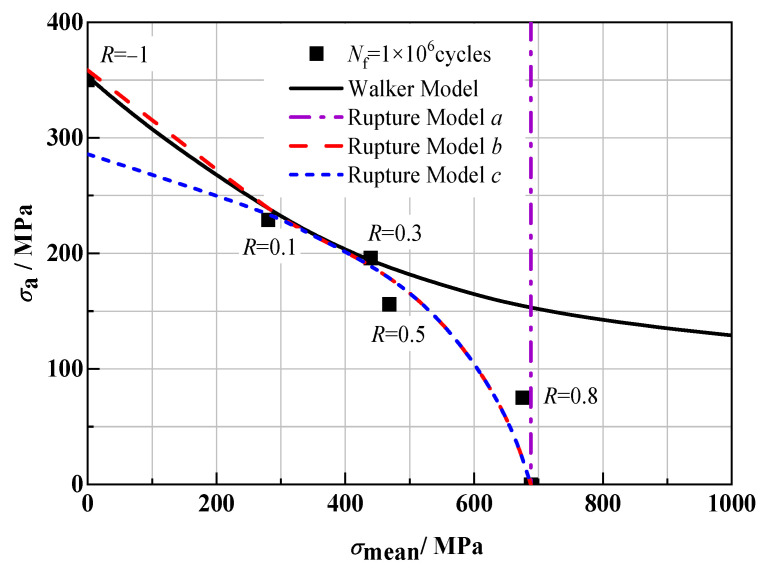
Equal life curve (*N*_f_ = 1 × 10^6^) of different life models.

Figure 8 also shows the equal life curve of the above model at 1 × 10^6^ cycles. The results show that the predictive capabilities presented by each model are consistent with those at 1 × 10^5^ cycles. It can be found that for the Walker Model, it can fit the test data points well under the condition of low stress ratio (*R* < 0.5), but when *R* > 0.5, the mixed mode with fatigue failure characteristics and tensile endurance fracture characteristics dominates, and the fitting results have a large deviation from the measured values. Rupture Model *a* has a good fitting effect under the condition of high stress ratio, but with the decrease in *R*, the fitting result deviates greatly from the measured value, and it cannot be used to predict the fatigue life under the condition of *R* < 0.8 at all. The predicted results of Rupture Model *b* are in good agreement with the measured values under different stress ratios. The fitting result of Rupture Model *c* is completely consistent with that of Rupture Model *b* under the condition of positive stress ratio, but under the condition of *R* < 0, the stress level of the fitting point is obviously low, and the fitting result is poor.

### 4.2. Improved Life Prediction Model and Prediction Results

#### 4.2.1. Improved Fatigue Life Prediction Model

Through the above analysis, it can be found that the above four models each have their own focuses in predicting life under different stress ratios, depending on the different damage modes. In fact, fatigue and tensile endurance are not two completely independent processes, but are mutually coupled. Wright et al. [36] found that under high-temperature conditions, a single crystal superalloy would experience an interaction of fatigue and creep during high-cycle fatigue tests, and proposed a hybrid model correlating fatigue and creep damage to predict the fatigue life of the alloy. Therefore, the fatigue and tensile endurance process in this paper were analyzed, and the high-cycle fatigue life under different stress ratios is modeled in a unified manner.

On this basis, the comprehensive influence of fatigue failure and time-dependent creep damage caused by mean stress is considered comprehensively, and the linear damage rule is adopted, namely:

1 = *D*_R_ + *D*_F_ = (*t*/*t*_r_) + (*N*/*N*_f_) (9)

If no coupling reaction occurs, such as in the case of pure tension, i.e., *R* = 1, or in the case of pure fatigue, i.e., *R* = −1, where the mean stress is zero, then the theoretical result should be *D*_R_ = 1 or *D*_F_ = 1.

By combining the Walker Model with the three Rupture Models *a*, *b*, and *c* as Equation (9), three composite models can be obtained: Composite Model *A* (Walker Model + Rupture Model *a*), Composite Model *B* (Walker Model + Rupture Model *b*) and Composite Model *C* (Walker Model + Rupture Model *c*). The four models mentioned in Section 4.1, which only considered a single fracture mode, and the above three composite models were used to simulate the fatigue data, and the fitting ability of each model to the test results was compared.

The predictive power of all models was compared according to the following three provisions:

(1) Calculate the total damage value *D*_total_ = *D*_R_ + *D*_F_ of different models. The closer the calculated total damage value *D*_total_ is to 1, the better the prediction effect will be.
(2) Calculate the standard deviation *s*_e_ of the model fitting results.

(10) $$s_{e}=\sqrt{\frac{\sum {\left[log\left(N_{f}^{i}\left(pred\right)\right)-log\left(N_{f}^{i}\left(obs\right)\right)\right]}^{2}}{M-P}}$$

In the formula, $N_{f}^{i}\left(pred\right)$ is the predicted cycle number from fatigue to fracture, $N_{f}^{i}\left(obs\right)$ is the cycle number of measured fatigue life, *M* is the number of samples, and *P* is the number of fitting parameters used in each model. The smaller the standard deviation *s*_e_, the better the prediction effect of the model.

(3) The predicted fatigue life is compared with the measured value. Under ideal conditions, the average value of the measured fatigue life at the same stress level under the same stress ratio is the same as the predicted value, and all test points are distributed around the predicted value.

#### 4.2.2. Fatigue Life Prediction by Single and Composite Models

Table 6 presents the standard deviation *s*_e_ and the average total damage value *D*_total_ of seven models. As for the Walker Model, the standard deviation *s*_e_ is relatively large, and there is a certain gap between *D*_total_ and 1, which means that its predictive ability for data point lifetime is poor. As for the Rupture Models, it can be seen from the table that for Rupture Model *a*, the standard deviation cannot be calculated, and its average total damage value is also far below 1, thus it can be excluded from the scope of investigation. The performance of Rupture Model *b* is better than Rupture Model *c* because of the slightly higher *s*_e_ and lower *D*_total_, which indicates that it has a good predictive ability for data point life. As for the composite models, Composite Model *A* has a *D*_total_ closest to 1, and its standard deviation *s*_e_ is also relatively small. Both Composite Model *B* and Composite Model *C* have a big difference between *D*_total_ and 1, and a higher standard deviation *s*_e_ than Composite Model *A*. Therefore, Composite Models *B* and *C* are excluded from the scope of this investigation.

**Figure 9:**
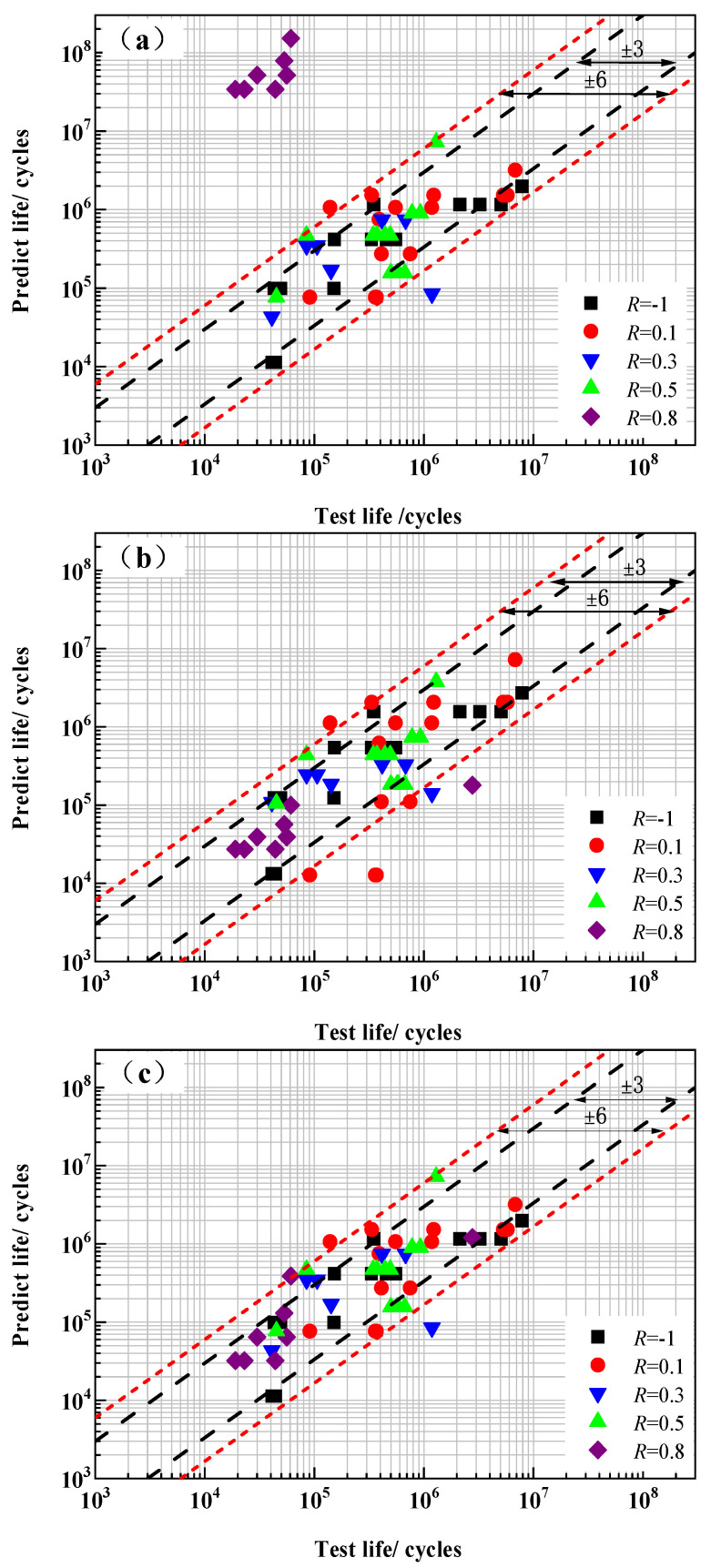
Comparison between the predicted fatigue life and experimental values. (**a**) Walker Model; (**b**) Rupture Model *b*; and (**c**) Composite Model *A*.

Therefore, after comprehensively considering the standard deviation *s*_e_ and the average total damage value *D*_total_, the Walker Model, Rupture Model *b*, and Composite Model *A* were selected for further detection. The comparison between the predicted fatigue life and the measured values of these three models is shown in Figure 9.

Figure 9a shows a comparison chart of the predicted fatigue life of the Walker Model with the measured values. It can be found from the figure that the model has a poor prediction effect on the test values under the condition of *R* = 0.8, and its predicted life is much higher than the measured values. It can be considered that under this stress ratio condition, creep damage caused by high mean stress significantly shortens the fatigue life of the samples and affects the prediction effect of the Walker Model under this condition.

Figure 9b shows the comparison between the predicted fatigue life of Rupture Model *b* and the measured values. It can be seen from the figure that part of the predicted data of Rupture Model *b* jumps out of the dispersion band of ±6, and its predicted data is relatively dispersed.

Figure 9c shows the comparison between the predicted fatigue life of Composite Model *A* and the measured value. It can be found from the figure that all the predicted data except one point are within the dispersion band of ±6, and more than 70% of the fatigue points are distributed within the dispersion band of ±3. By comparing Figure 9b with Figure 9c, the prediction effect of Composite Model *A* is better than that of Rupture Model *b*, and Composite Model *A* has the best fatigue life prediction ability.

## 5. Conclusions

(1) At 400 °C, the high-cycle fatigue data of the SLM Ti6Al4V alloy are relatively dispersed, especially under conditions of low stress ratios.
(2) The fracture mechanism has been revealed. As the number of cycles increases, fatigue cracks mainly originate from the pore defects existing on the surface or subsurface of the sample due to stress concentration. Subsequently, the crack enters a stable expansion stage with the fracture surface flat and the fatigue bands visible. As the crack continues to expand, the specimen enters an unstable stage, rapidly expanding to fracture. The fracture surface is accompanied by dimples, and this is known as a ductile fracture.
(3) Through the research on the fracture, it is found that at 400 °C, SLM Ti6Al4V alloy exhibits a failure mode characterized by a mixed effect of fatigue failure and tensile endurance fracture. With the increase in the stress ratio, the proportion of fatigue failure influence gradually decreases, while the proportion of creep damage influence gradually increases.
(4) Aiming at the prediction of high-cycle fatigue life of the SLM Ti6Al4V alloy at 400 °C, a life model considering fatigue failure and creep damage is proposed. The composite model is used to predict the high-cycle fatigue life uniformly under different stress ratios, and the predicted results are in good agreement with the test results.

## Figures and Tables

**Table 1:** Normal and actual chemical compositions of Ti6Al4V powders [11].

[wt/%]	Al	V	Fe	O	C	N	H	Y	Si	Ti
Standard value	5.6~6.5	3.4~4.5	≤0.25	0.08~0.13	≤0.05	≤0.03	≤0.0125	≤0.005	≤0.1	balance
Actual value	6.29	3.99	0.2	0.12	0.009	0.02	0.002	<0.005	0.031	balance

**Table 2:** Tensile properties of SLM Ti6Al4V alloy at 400 °C [29].

Temperature/°C	Orientation	UTS/MPa	YS/MPa	Elongation/%	Reduction in Area/%
400	Vertical	688	545	16	65
	Horizontal	742	619	15	62

**Table 3:** The fitting parameters of the *S*-*N* curve at 400 °C.

Stress Ratio	*B*_1_	*B*_2_	*B*_3_	Correlation Coefficient
−1	30.68	−10.53	134.33	0.794
0.1	28.56	−9.79	308.35	0.757
0.3	53.57	−17.12	-	0.662
0.5	57.12	−17.94	-	0.622
0.8	133.69	43.82	-	0.865

**Table 4:** Walker Model fitting parameters.

	*A*_1_	*A*_2_	*w*	Correlation Coefficient
Parameter values	55.63	−9.02	−18.42	0.714

**Table 5:** Fitting parameters of Rupture Model *a*, *b* and *c*.

Rupture Model	lg*K*_r_	*n*	Correlation Coefficient
*a*	211.71	−30.78	0.112
*b*	36.12	−13.15	0.774
*c*	37.86	−13.79	0.767

**Table 6:** Comparison of prediction ability of life model.

Model	$\mathrm{Standard}\;\mathrm{Deviation}\;s_{e}$	$\mathrm{Average}\;\mathrm{Total}\;\mathrm{Damage}\;\mathrm{Value}\;D_{total}$
Walker Model	1.409	1.251
Rupture Model *a*	large	0.172
Rupture Model *b*	0.696	0.985
Rupture Model *c*	0.700	0.977
Composite Model *A*	0.726	1.010
Composite Model *B*	0.731	2.413
Composite Model *C*	0.742	2.563

## Data Availability

The original contributions presented in the study are included in the article; further inquiries can be directed to the corresponding authors.

## Appendix A

Rupture Models *b* and *c* calculate the influence of alternating stress components and mean stress components on a fracture by integrating the fracture damage during the test process. By dividing the entire cycle into small time increments, it is considered that within the infinitely small time increments, the alternating stress component and the average stress component are constant values, causing accumulable damage and leading to the final fracture.

For the load waveform of a sine wave, there is

*σ*(*t*) = *σ*_mean_ + *σ*_a_·sin(2π*t*/*τ*) (A1)

When the fracture time is *t*_r_, the cumulative damage of the final fracture *D*_R_ should be 1 at the expected service life *τ*:

(A2) $$D_{R}=1=\int_{0}^{\tau }dt/t_{r}\left(\sigma \left(t\right)\right)$$

For the Rupture Model *b*, the integration interval of *t* is [0,*τ*]. Within the integral interval, it is considered that within the time increment *dt*, the relationship between *t*_r_ and *σ*(*t*) is:

(A3) $$t_{r}=K_{r}{\left[\sigma \left(t\right)\right]}^{m}$$

Substituting this into Equation (A2), we can obtain

(A4) $$1=\int_{0}^{\tau }dt/t_{r}\left(\sigma \left(t\right)\right)=\int_{0}^{\tau }dt/K_{r}{\left[\sigma_{mean}+\sigma_{a}\sin\left(2\pi t/\tau \right)\right]}^{m}$$

Let 2π*t*/*τ* = *x*, then d*x* = (2π/*τ*)∙*dt*, and the integration interval is *x* ∈ [0, 2π]

(A5) $$1=\int_{0}^{2\pi }\left\{\left(\tau /2\pi \right)/\left\{K_{r}{{\left(\sigma_{max}/2\right)}^{m}\left[1+R+\left(1-R\right)\sin x\right]}^{m}\right\}\right\}dt$$

(A6) $$K_{r}\cdot \frac{2\pi }{\tau }{\left(\sigma_{max}/2\right)}^{m}=\int_{0}^{2\pi }\left\{1/{\left[1+R+\left(1-R\right)\sin x\right]}^{m}\right\}dt$$

The integral of the above formula is solved by the numerical calculation method. The Newton–Cotes product formula is used, and its integration formula is:

(A7) $$\int_{a}^{b}f\left(x\right)dx=\sum_{k=0}^{n}c_{k}^{\left(n\right)}f\left(a+k\frac{b-a}{n}\right)$$

In the formula, [*a*,*b*] are the integration intervals, $x_{k}=a+k\frac{b-a}{n}$ are the equidistant integration nodes, and $c_{k}^{\left(n\right)}$ is the Cotes coefficient.

Then, to solve Equation (A6), the integration interval can be equally divided into eight segments, with a total of nine integration nodes, which are in sequence:

$$x_{0}=0,\;x_{k}=\frac{\pi }{4}\cdot k\left(k=0,1,2,\ldots ,8\right)$$

The Cotes coefficient is:

**Table A1:** The Cotes coefficient.

k	0	1	2	3	4	5	6	7	8
*c*_k_	$\frac{989}{28350}$	$\frac{5888}{28350}$	$\frac{-928}{28350}$	$\frac{10496}{28350}$	$\frac{-45440}{28350}$	$\frac{10496}{28350}$	$\frac{-928}{28350}$	$\frac{5888}{28350}$	$\frac{989}{28350}$

We can get:

(A8) $$K_{r}\cdot \frac{2\pi }{\tau }{\left(\sigma_{max}/2\right)}^{m}\approx \sum_{k=0}^{8}c_{k}\frac{1}{{\left[1+R+\left(1-R\right)\sin x_{k}\right]}^{m}}$$

(A9) $$K_{r}\cdot 2\pi \cdot {\left\{\sum_{k=0}^{8}c_{k}\frac{1}{{\left[1+R+\left(1-R\right)\sin x_{k}\right]}^{m}}\right\}}^{-1}\cdot {\left(\sigma_{max}/2\right)}^{m}=\tau$$

Let $M=2\pi \cdot {\left\{\sum_{k=0}^{8}c_{k}\frac{1}{{\left[1+R+\left(1-R\right)\sin x_{k}\right]}^{m}}\right\}}^{-1}$, then

(A10) $$K_{r}\cdot M\cdot {\left(\sigma_{max}/2\right)}^{m}=\tau$$

*K*_r_ is obtained by using the linear regression equation. The dichotomy method is adopted to compute the *m* value to make the standard deviation $s_{e}=\sqrt{\frac{\sum {\left[log\left(N_{f}^{i}\left(pred\right)\right)-log\left(N_{f}^{i}\left(obs\right)\right)\right]}^{2}}{M\;-P}}$ minimum.

Then, the fitting formula can be obtained:

(A11) $$t_{r}=K_{r}{\left[\bar{\sigma }\left(t\right)\right]}^{m}=K_{r}\cdot M\cdot {\left(\sigma_{max}/2\right)}^{m}$$

For Rupture Model *c*, its calculation process in the case of *R* > 0 is consistent with that of Fracture Model *b*. For the case where *R* < 0, the situation when the stress is compressive stress needs to be ignored. When *R* = −1, under the condition of ignoring the influence of compressive stress, the integral interval becomes *x* ∈ [0, π].
